# Human neural stem cells derived from fetal human brain communicate with each other and rescue ischemic neuronal cells through tunneling nanotubes

**DOI:** 10.1038/s41419-024-07005-w

**Published:** 2024-09-01

**Authors:** D. L. Capobianco, R. De Zio, D. C. Profico, M. Gelati, L. Simone, A. M. D’Erchia, F. Di Palma, E. Mormone, P. Bernardi, A. Sbarbati, A. Gerbino, G. Pesole, A. L. Vescovi, M. Svelto, F. Pisani

**Affiliations:** 1 Department of Biosciences, Biotechnologies and Environment, University of Bari “Aldo Moro”, Bari, Italy; 2 Fondazione IRCCS Casa Sollievo della Sofferenza, San Giovanni, Rotondo, Foggia, Italy; 3 Institute of Biomembranes, Bioenergetics and Molecular Biotechnologies (IBIOM) CNR, Bari, Italy; 4 Department of Neurosciences, Biomedicine and Movement Sciences. Unit of Human Anatomy, University of Verona, Verona, Italy; 5 Faculty of Medicine, Link Campus University, Rome, Italy; 6 National Institute of Biostructures and Biosystems (INBB), Rome, Italy

**Keywords:** Neural stem cells, Neurophysiology

## Abstract

Pre-clinical trials have demonstrated the neuroprotective effects of transplanted human neural stem cells (hNSCs) during the post-ischemic phase. However, the exact neuroprotective mechanism remains unclear. Tunneling nanotubes (TNTs) are long plasma membrane bridges that physically connect distant cells, enabling the intercellular transfer of mitochondria and contributing to post-ischemic repair processes. Whether hNSCs communicate through TNTs and their role in post-ischemic neuroprotection remains unknown. In this study, non-immortalized hNSC lines derived from fetal human brain tissues were examined to explore these possibilities and assess the post-ischemic neuroprotection potential of these hNSCs. Using Tau-STED super-resolution confocal microscopy, live cell time-lapse fluorescence microscopy, electron microscopy, and direct or non-contact homotypic co-cultures, we demonstrated that hNSCs generate nestin-positive TNTs in both 3D neurospheres and 2D cultures, through which they transfer functional mitochondria. Co-culturing hNSCs with differentiated SH-SY5Y (**d**SH-SY5Y) revealed heterotypic TNTs allowing mitochondrial transfer from hNSCs to **d**SH-SY5Y. To investigate the role of heterotypic TNTs in post-ischemic neuroprotection, **d**SH-SY5Y were subjected to oxygen-glucose deprivation (OGD) followed by reoxygenation (OGD/R) with or without hNSCs in direct or non-contact co-cultures. Compared to normoxia, OGD/R **d**SH-SY5Y became apoptotic with impaired electrical activity. When OGD/R **d**SH-SY5Y were co-cultured in direct contact with hNSCs, heterotypic TNTs enabled the transfer of functional mitochondria from hNSCs to OGD/R **d**SH-SY5Y, rescuing them from apoptosis and restoring the bioelectrical profile toward normoxic **d**SH-SY5Y. This complete neuroprotection did not occur in the non-contact co-culture. In summary, our data reveal the presence of a functional TNTs network containing nestin within hNSCs, demonstrate the involvement of TNTs in post-ischemic neuroprotection mediated by hNSCs, and highlight the strong efficacy of our hNSC lines in post-ischemic neuroprotection.

**Figure:**
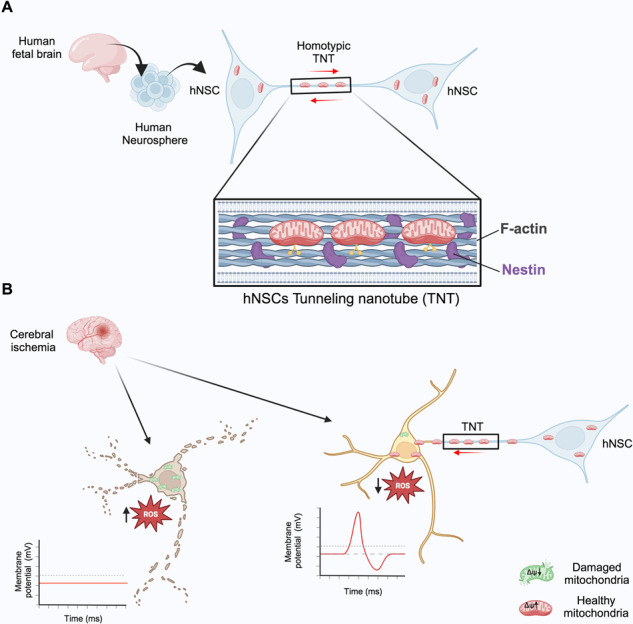
Human neural stem cells (hNSCs) communicate with each other and rescue ischemic neurons through nestin-positive tunneling nanotubes (TNTs). **A** Functional mitochondria are exchanged via TNTs between hNSCs. **B** hNSCs transfer functional mitochondria to ischemic neurons through TNTs, rescuing neurons from ischemia/reperfusion ROS-dependent apoptosis.

Human neural stem cells (hNSCs) communicate with each other and rescue ischemic neurons through nestin-positive tunneling nanotubes (TNTs). **A** Functional mitochondria are exchanged via TNTs between hNSCs. **B** hNSCs transfer functional mitochondria to ischemic neurons through TNTs, rescuing neurons from ischemia/reperfusion ROS-dependent apoptosis.

## Introduction

Neural stem cells (NSCs) are self-renewing, multipotent cells capable of differentiating into neurons, astrocytes, and oligodendrocytes [1, 2]. Discovered in 1992 [3], they now play a crucial role in cutting-edge regenerative medicine approaches for treating neurodegenerative diseases [4–10].

Preclinical studies in murine models of cerebral ischemia have demonstrated that engrafted NSCs in the post-ischemic phase improve stroke outcomes by promoting angiogenesis and neurogenesis, reducing vascular inflammation, restoring the blood-brain barrier, and enhancing neuron functions [10–14]. Notably, human NSCs (hNSCs) have exhibited neuroprotective effects in murine models, rescuing neurons from ischemia-induced apoptosis without differentiating into neurons [15]. This neuroprotection seems to be related to the secretion of neuroprotective signals by hNSCs, such as immunomodulating molecules and trophic and growth factors, along with the activation of anti-apoptotic pathways, a mechanism known as the “paracrine hypothesis” [16–20]. In this context, long-range intercellular signal transfer mediated by hNSC-derived extracellular vesicles, which can transport ions, proteins, nucleic acids, and organelles, has been shown to participate in the crosstalk between hNSCs and between hNSCs and host tissues [21–24].

However, the exact mechanisms through which hNSCs communicate with each other and enhance neuron function after an ischemic stroke are still largely unknown. Clarifying the new ways in which this occurs could contribute to the design of novel and more effective post-ischemic therapeutic strategies.

Recently, a new long-range and contact-dependent intercellular communication mechanism based on tunneling nanotubes (TNTs) has emerged as a pivotal player in physiological long-range intercellular communication and tissue repair processes. TNTs are long membranous protrusions containing F-actin that physically connect distant cells and have been observed both in vitro and in vivo [25–30]. TNTs facilitate the transfer of cellular cargoes, including macromolecules such as proteins and nucleic acids, as well as organelles, between cells of the same type (homotypic TNTs) and between cells of different types (heterotypic TNTs). This creates a cellular network that helps to regulate tissue functions.

TNTs play a particularly significant role in the intercellular transfer of functional mitochondria, which contributes to rescuing recipient cells from bioenergetic deficiencies and apoptosis induced by pathological conditions affecting mitochondria activity [31–34]. Ischemic stroke is one of the most prevalent pathological conditions affecting mitochondrial activity and with a severe impact on the patient’s health. Current therapies, while effective to some extent, often trigger ischemia-reperfusion (IR) injury. In the IR injury the reactive oxygen species (ROS) strongly increases, this induces the opening of mitochondrial permeability transition pore [35, 36] and Ca^2+^ accumulation. This decreases mitochondrial membrane potential and induces mitochondrial swelling, promoting activation of the mitochondrial apoptosis pathway, caspase activation and neuron apoptosis, exacerbating the injury [37–43].

In this context, the transfer of healthy mitochondria via TNTs has been proposed as a promising therapy [44–47]. This approach has been mainly explored with mesenchymal stem cells (MSCs), which generate heterotypic TNTs with ischemic receiving cells to rescue them from ischemia-induced apoptosis by transferring functional mitochondria [33, 48–51].

To date, it is unknown whether human neural stem cells communicate with each other and with human neurons through TNTs and whether this mechanism plays a role in the post-ischemic protection of human neurons.

To address these questions, we analyzed non-immortalized human neural stem cell lines isolated from brain tissue samples extracted from fetuses that died due to spontaneous abortion. These cells have undergone a rigorous selection, isolation, and expansion process, culminating in the final pharmaceutical formulation [52].

Here, our aim is to investigate whether TNTs-based intercellular communication occurs between hNSCs and between hNSCs and human neuronal cells, and whether TNTs play a role in hNSC-based neuroprotection of ischemic neuronal cells. Furthermore, in this study, we evaluate the efficacy of our non-immortalized hNSCs in rescuing ischemic neuronal cells in vitro.

Our results reveal the capacity of these cells to communicate with each other via F-actin and nestin-positive TNTs, through which they transfer functional mitochondria. In heterotypic co-cultures with normal differentiated SH-SY5Y (**d**SH-SY5Y), we demonstrate the ability of these cells to transfer functional mitochondria to human **d**SH-SY5Y through TNTs. Using an in-vitro model of ischemia/reperfusion, we show that our hNSCs significantly protect human **d**SH-SY5Y from ROS-dependent apoptosis induced by ischemia/reperfusion only in the presence of direct contact in which we observe the TNTs-mediated mitochondria transfer from hNSCs to ischemic **d**SH-SY5Y. More importantly, hNSCs almost completely restore neuron plasma membrane resting potential, plasma membrane evoked current and evoked action potentials of ischemic **d**SH-SY5Y when in direct contact with hNSCs.

Our findings offer a new perspective on the contact-dependent mechanisms of communication between hNSCs, as well as between hNSCs and **d**SH-SY5Y. Additionally, they highlight the effectiveness of our hNSCs in rescuing ischemic **d**SH-SY5Y from post-ischemic insult, emphasizing the quality of the hNSCs-based pharmaceutical formulation [52] and the role of TNTs-mediated crosstalk in this mechanism.

## Results

### Nestin-positive TNTs-like structures connect hNSCs within neurospheres

**Fig. 1:**
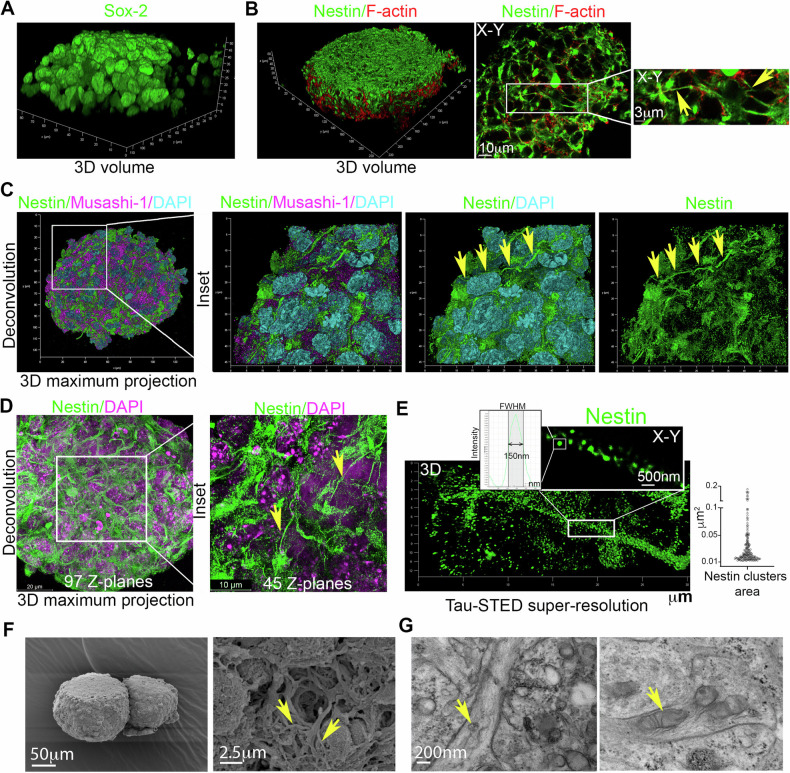
Nestin-positive TNT-like structures inside neurospheres and nestin supramolecular assembly state inside TNTs. **A** and **B** Localization of Sox-2 (**A**), F-actin (red) with Nestin (green) (**B**) in 60 μm-thick neurosphere sections analyzed using laser-scanning confocal microscopy, followed by 3D reconstruction and *X*–*Y*–*Z* slicing analysis. A single *X*–*Y* plane within the neurosphere is displayed (**B**, right). The zoomed-in box highlights nestin-positive TNTs-like structures (indicated by yellow arrows). Representative images were obtained from three different hNSCs donors. **C** Localization of nestin (green), Musashi-1 (magenta), and DAPI (cyano) in 60 μm-thick neurosphere sections analyzed via laser-scanning confocal microscopy, followed by 3D reconstruction. A 3D maximum projection is presented. The inset indicates the presence of nestin-positive TNTs-like structures connecting Musashi-1-positive cells. Representative images were obtained from three different hNSC donors. **D** 3D maximum projection of 97 *z*-planes displaying nestin localization in 60 μm-thick neurosphere sections analyzed using laser-scanning confocal microscopy, followed by 3D reconstruction. These nestin-positive connections form an extensive network that spans the entire volume of the neurosphere. Representative images were obtained from three different hNSC donors. **E** Nestin localization in neurospheres analyzed through Tau-STED super-resolution confocal microscopy. Discrete nestin supramolecular assemblies are visible. Analysis of nestin cluster dimensions reveals nestin assemblies within 0.01 and 0.05 μm^2^. Data were obtained using three different hNSC donors. **F** and **G**. SEM images at high resolution show the protrusions (arrows) on the neurospheres area (**F**). TEM images show the presence of elongated mitochondria (arrows) inside the protrusions (**G**).

Human neurospheres were sliced into 60 μm-thick sections and immunostained for Sox-2 (Fig. 1A), Musashi-1, Nestin, and F-actin. Confocal analysis of whole sections revealed the presence of long nestin-positive TNTs-like structures connecting distant hNSCs (Fig. 1B, C). The 3D maximum projection of all 97 Z-planes shows that these connections create an extensive network that spans the entire volume of the neurosphere (Fig. 1D). Super-resolution imaging using Tau-STED revealed the assembly state of nestin inside these TNTs-like structures, which appeared as polygonal structures with major frequent area ranging from 0.01 to 0.05 μm^2^ (Fig. 1E).

Furthermore, human neurospheres were analyzed by scanning electron microscopy (SEM), confirming the presence of long cell protrusions, with a diameter compatible with TNTs (Fig. 1F). Further analysis by transmission electron microscopy (TEM) confirms the presence of numerous large and elongated mitochondria inside the protrusions, among other organelles (Fig. 1G).

**Fig. 2:**
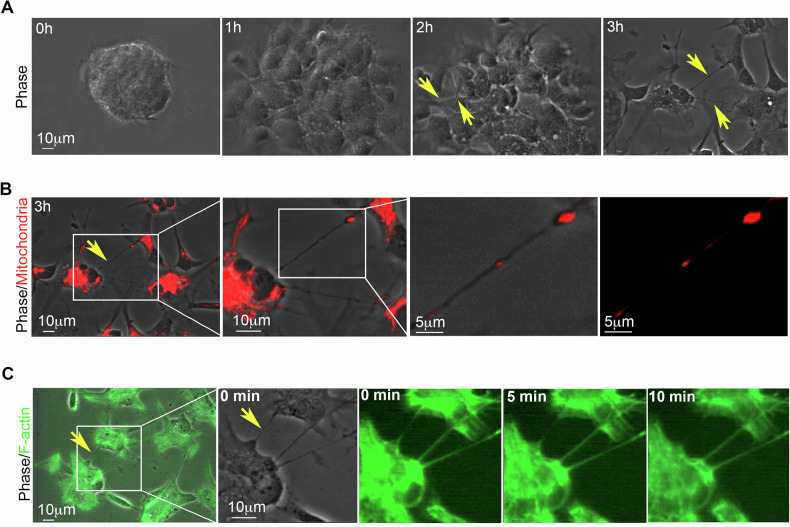
TNTs-like structures during neurosphere migration. **A** A migrating neurosphere was analyzed using time-lapse phase contrast microscopy. Highly dynamic TNTs-like structures were clearly visible starting two hours after seeding (yellow arrows). Representative images were obtained from three different hNSC donors. **B** and **C** Three hours after seeding, a migrating neurosphere was stained for mitochondria and F-actin and analyzed using live-cell time-lapse fluorescence microscopy. Highly dynamic TNTs-like structures, containing functional mitochondria (**B**) and F-actin (**C**), were present during neurosphere migration (yellow arrows). Supplementary Videos 1–4. Representative images were obtained from three different hNSC donors.

To investigate whether TNTs-like structures appear during hNSC migration, integral and live neurospheres were seeded onto cultrex-coated glass and analyzed using live-cell time-lapse microscopy. Phase-contrast imaging clearly depicted the presence of highly dynamic TNTs-like structures during neurosphere migration (Fig. 2A, Supplementary Videos 1, 2). Migrating neurospheres were stained for mitochondria and F-actin and then analyzed using live-cell fluorescence microscopy 3 h after seeding. The data indicated the presence of mitochondria (Fig. 2B) and F-actin (Fig. 2C, Supplementary Videos 3, 4) within these highly dynamic TNTs-like structures connecting distant hNSCs during neurosphere migration.

These findings demonstrate that hNSCs are interconnected through nestin-positive TNTs-like structures within the neurosphere. These connections form an extensive network within the neurosphere volume and persist during neurosphere migration.

### Nestin supramolecular clusters localize inside functional TNTs between hNSCs

**Fig. 3:**
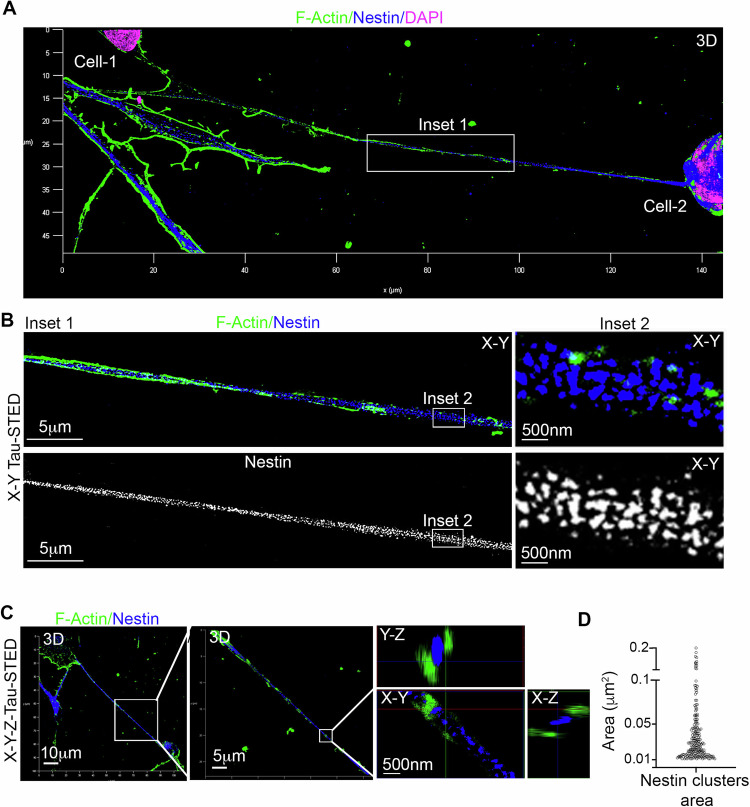
Tau-STED super-resolution microscopy reveals nestin assembly inside TNTs. **A** Human neural stem cells (hNSCs) in 2D culture were stained for F-Actin (green) and Nestin (blue), with nuclei labeled using DAPI (pink). hNSCs formed connections through elongated F-actin-rich TNTs, which also displayed Nestin positivity (3D reconstruction created from deconvoluted *X*–*Y*–*Z* confocal planes). Representative images were obtained from three different hNSC donors. **B** To obtain a more detailed view of the region marked as inset 1 in panel **A**, we employed Tau-STED super-resolution microscopy to examine a single *X*–*Y* plane. In inset 2, at a higher magnification, discernible and organized nestin structures are clearly visible within discrete clusters. Representative images were obtained from three different hNSC donors. **C** Utilizing 3D *X*–*Y*–*Z* Tau-STED microscopy, we investigated TNTs connecting hNSC cells. *Z*-projections illustrate the spatial distribution of F-actin and nestin within TNTs. F-actin and nestin do not co-localize within TNTs. **D** Tau-STED analysis distinctly demonstrates the presence of supramolecular nestin clusters ~0.01–0.05 μm^2^ area inside TNTs. Representative images and data were obtained from three different hNSC donors.

To thoroughly investigate the intercellular communication mediated by TNTs among hNSC cells, it is essential to analyze hNSCs in 2D culture conditions. For this purpose, human neurospheres were mechanically disaggregated and then seeded as a single-cell suspension on a cultrex-coated glass surface. To specifically examine TNTs, cells were subjected to immunofluorescence analysis for Sox2, Nestin, and F-actin localization, followed by confocal microscopy and 3D reconstruction, as well as 3D Tau-STED super-resolution microscopy. Confocal *X*–*Y* planes were specifically analyzed for TNT-specific morphometric properties, such as detachment from the substrate, positivity for F-actin, and straightness [26]. Our data unequivocally reveal the presence of F-actin-positive TNTs, and notably, these TNTs were also found to be highly positive for nestin (Supplementary Fig. 1A–C). Measuring the diameters of TNTs using the full width at half-maximum (FWHM) analysis, both TNT-specific F-actin and nestin signals were found to be in the range of 400–700 nm in diameter (Supplementary Fig. 1D). To delve deeper into the assembly of nestin within TNTs, samples were analyzed using *X*–*Y* Tau-STED super-resolution microscopy. *X*–*Y* Tau-STED analysis distinctly demonstrates the presence of supramolecular nestin clusters ~0.01–0.05 μm^2^ area inside TNTs (Fig. 3A, B), resembling those observed in intact neurospheres. Using *X*–*Y*–*Z* Tau-STED analysis followed by 3D-reconstruction and *Z*-projections, we demonstrated that nestin clusters localize between F-actin fibers like a “bridge”.

**Fig. 4:**
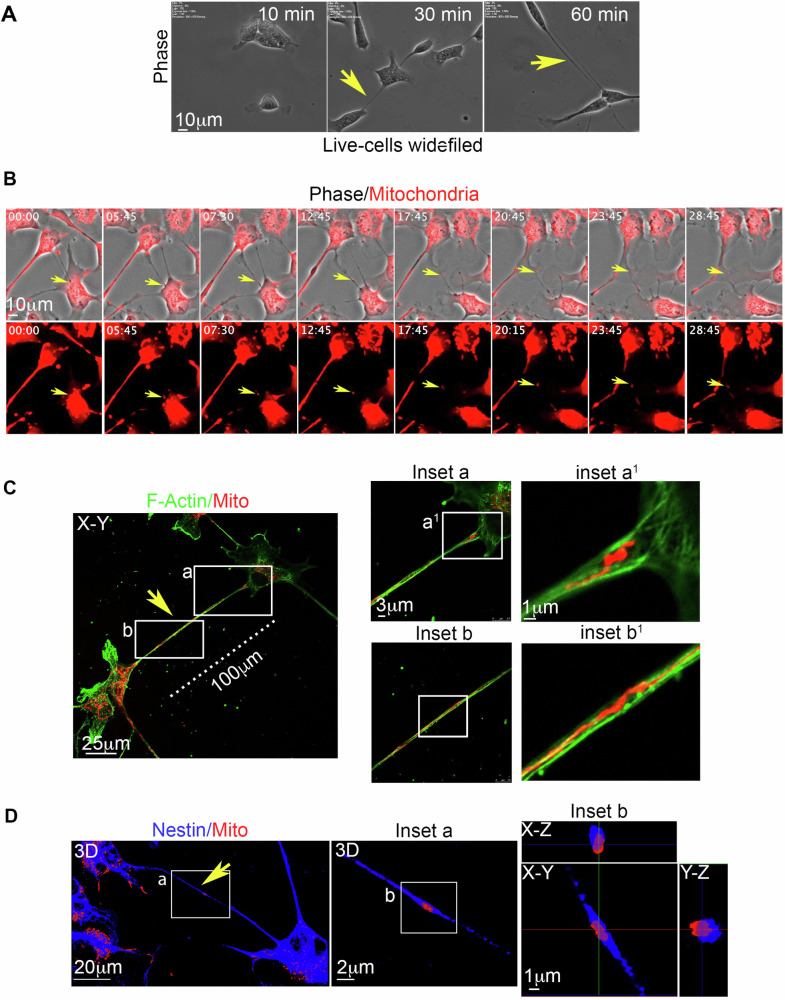
Mitochondrial transport in F-actin and nestin-positive functional TNTs between hNSCs. **A** Live-cell time-lapse widefield phase-contrast microscopy reveals that hNSCs rapidly generate TNTs when cultured in 2D. **B** hNSCs were stained with ∆Ψ-dependent MitoTracker Deep Red and analyzed using live-cell time-lapse fluorescence microscopy. The presence of functional mitochondria traveling through TNTs between hNSCs is indicated (Supplementary Videos 5–7). **C** and **D** hNSCs were stained with ∆Ψ-dependent MitoTracker Deep Red and analyzed through laser-scanning confocal microscopy, along with *X*–*Y* and *Z*-projection analysis. F-actin-positive (**C**) and Nestin-positive (**D**) TNTs, in which functional mitochondria were observed, are highlighted in zoomed insets. Representative images were obtained from three different hNSC donors.

Next, we explored the dynamic properties of these TNTs and the dynamics of mitochondria within TNTs in 2D culture, another property of functional TNTs. For this purpose, hNSCs were seeded as a single-cell suspension at low density on a cultrex-coated glass surface, and mitochondria were stained. Live-cell time-lapse fluorescence analysis revealed that hNSCs generated highly dynamic TNTs (Fig. 4A), within which mitochondria were transported (Fig. 4B, Supplementary Videos 5–7). Subsequently, cells were fixed, stained for Nestin and F-actin, and subjected to confocal microscopy analysis. The data unambiguously demonstrate the presence of mitochondria within F-actin-positive and nestin-positive TNTs (Fig. 4C, D).

### Nestin-positive TNTs mediate the intercellular transfer of mitochondria between hNSCs

**Fig. 5:**
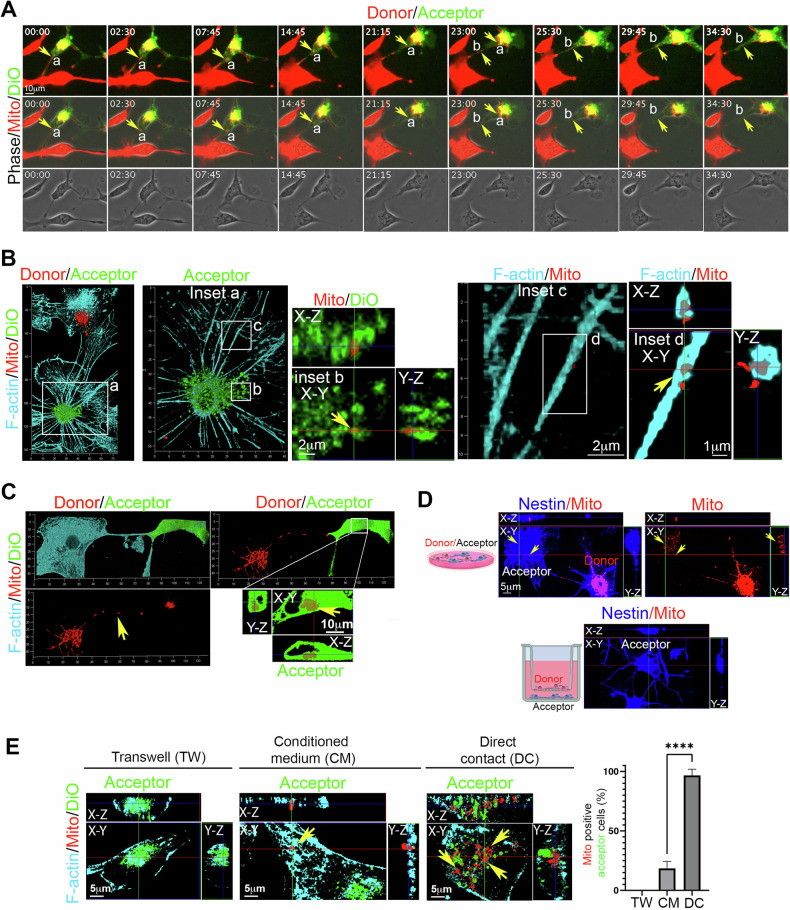
TNT-mediated intercellular transfer of functional mitochondria between hNSCs. **A** hNSCs were stained with ΔΨ-dependent MitoTracker Deep Red and co-cultured in direct contact with acceptor hNSCs stained with DiO. Live-cell time-lapse fluorescence microscopy revealed functional mitochondria traveling through TNTs from donor to acceptor hNSCs, as indicated by the yellow arrows (Supplementary videos 8–10). Representative images were obtained from three different hNSC donors. **B** and **C** The co-culture described in **A** was fixed, stained for F-actin (**B** and **C**) or Nestin (**D**), and analyzed using laser scanning confocal microscopy. This was followed by 3D reconstruction and *X*–*Y* and *Z*-projection analysis. The 3D reconstruction and slicing analysis of the DiO-acceptor cell clearly demonstrated the presence of mitochondria originating from the donor hNSC inside the acceptor DIO-positive hNSC. Representative images were obtained from three different hNSC donors. **D** hNSCs were stained with ΔΨ-dependent MitoTracker Deep Red and co-cultured without direct contact with acceptor hNSCs using the Transwell system. Donor cells were seeded on the Transwell, while acceptor cells were analyzed for Nestin and mitochondria localization using laser scanning confocal microscopy. This was followed by 3D reconstruction and *X*–*Y* and *Z*-projection analysis. Notably, there was a complete absence of mitochondria inside acceptor cells when the co-culture was not in direct contact. **E** The intercellular transfer of functional mitochondria was evaluated using non-contact transwell-based coculture (TW), conditioned medium (CM), or direct contact (DC). Donor hNSCs were stained with ΔΨ-dependent MitoTracker Deep Red (red), acceptor hNSCs were stained with DiO (green), and F-actin staining (cyan) was performed using a cell-permeable CellMask actin, avoiding cell permeabilization. The number of receiving cells containing mitochondria was measured in TW, CM, and DC. Only the direct contact coculture allows a robust transfer of mitochondria from donor to acceptor hNSCs (yellow arrows), while CM allows only rare and isolated mitochondrial signals inside receiving cells. Data are presented as mean percentage ± SEM. Data was obtained using three different hNSC donors. ****p < 0.0001. Representative images were obtained from three different hNSC donors.

To investigate the intercellular transfer of organelles, another primary property of bona-fide TNTs between hNSCs, we established a homotypic co-culture system based on hNSCs [26] (donor-receiving) and analyzed it using live-cell time-lapse fluorescence microscopy and 3D laser-scanning confocal microscopy (3D-LSCM). In this setup, donor hNSCs were stained with fixable mitochondrial membrane potential (Δψ)-dependent MitoTracker Deep Red, extensively washed after 24 h to remove excess dye and subsequently co-cultured with receiving hNSCs, stained with DiO. The co-culture was analyzed 24 h later. Live-cell time-lapse fluorescence microscopy clearly revealed mitochondria swiftly traversed TNTs from a donor cell to an acceptor hNSC (Fig. 5A. Supplementary videos 8–10). Following co-culture, the cells were fixed and analyzed for F-actin and nestin localization through 3D-LSCM. The 3D reconstructions and *X*–*Y*–*Z* slicing analysis substantially confirmed the capability of functional mitochondria to localize inside F-actin-positive TNTs and the transfer of functional mitochondria from donor hNSCs to receiving hNSCs (Fig. 5B, C).

To assess the potential contribution of TNT-independent mitochondria intercellular transfer [53], and to exclude possible technical issue due to dye passive diffusion [54], we established a non-contact co-culture system utilizing the Transwell® system (TW). After 24 h, cells were analyzed by immunofluorescence for nestin and mitochondrial localization. Nestin-positive TNTs connecting donor and acceptor cells containing mitochondria were clearly observed, and no signs of MitoTracker Deep Red were found in receiving cells in the TW-based coculture (Fig. 5D). Since cell permeabilization, necessary for immunofluorescence, could affect residual mitochondrial signals inside receiving cells in TW, and since TW porosity could prevent the transfer of vesicles containing mitochondria, we used TW and conditioned medium (CM) derived from hNSCs stained with MitoTracker Deep Red. F-actin staining was performed using a cell-permeable CellMask actin, avoiding cell permeabilization. The number of DiO-receiving cells containing mitochondria was measured in TW, CM, and direct contact (DC) setups. Results show that only the direct contact coculture allows a robust transfer of mitochondria from donor to acceptor hNSCs, while CM allows only rare and isolated mitochondrial signals inside receiving cells (Fig. 5E).

Collectively, these data underscore the ability of hNSCs to establish connections with one another through F-actin- and nestin-positive TNTs and that these TNTs are able to transfer functional mitochondria from one hNSCs to another hNSCs.

### hNSCs rescue ischemic dSH-SY5Y from ROS-induced apoptosis through TNTs-mediated transfer of functional mitochondria

We investigated whether hNSCs form heterotypic TNTs with **d**SH-SY5Y and whether these TNTs transfer functional mitochondria from hNSCs to normal **d**SH-SY5Y. To do this, we established both direct contact and non-contact co-cultures. hNSCs were stained with fixable ΔΨ-dependent MitoTracker Deep Red and co-cultured with human **d**SH-SY5Y either in direct contact or in non-contact coculture. After 24 h, active mitochondria from hNSCs were found inside **d**SH-SY5Y only in the direct contact co-culture (Supplementary Fig. 2A). A more detailed confocal analysis revealed TNTs connecting hNSCs and **d**SH-SY5Y in which functional mitochondria were clearly present, along with hNSCs’ mitochondria inside **d**SH-SY5Y (Supplementary Fig. 2B).

Given that TNTs-mediated intercellular transfer of functional mitochondria can rescue post-ischemic neural cells from apoptosis triggered by mitochondrial dysfunctions, we investigated whether this phenomenon occurs between healthy hNSCs and post-ischemic **d**SH-SY5Y.

**Fig. 6:**
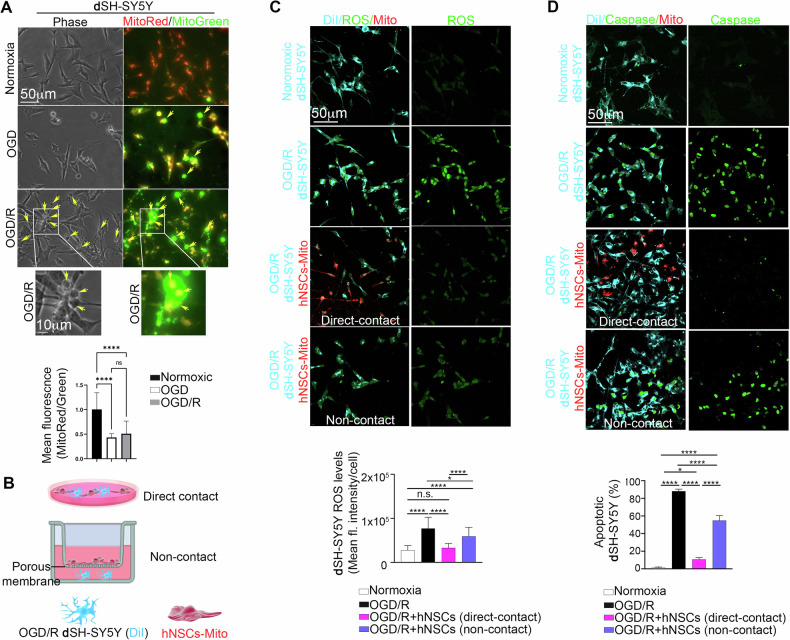
Direct contact is essential for the complete rescue of post-ischemic dSH-SY5Y from ROS-triggered apoptosis. **A** Normoxic, OGD, and OGD/R differentiated SH-SY5Y (**d**SH-SY5Y) were stained with Mitotracker Green AM, indicating mitochondria mass, and ΔΨ-dependent MitoTracker Red CMXRos, which selectively stained active and functional mitochondria. The Mitotracker Red/Green fluorescent ratio served as a semiquantitative analysis of mitochondria activity. OGD and OGD/R strongly affect mitochondria activity. OGD/R induces highly frequent changes in cell morphology in dSH-SY5Y, suggesting an apoptosis process (yellow arrows). ****p < 0.0001. **B** hNSCs were stained with fixable ΔΨ-dependent MitoTracker Deep Red (red) and cocultured in direct contact or in non-contact with human **d**SH-SY5Y stained with DiI and subject to oxygen-glucose deprivation (OGD/R). **C** Dil-labeled human **d**SH-SY5Y (Cyano) were cultured in normoxic conditions or exposed to OGD, washed, and reoxygenated (OGD/R) with or without a constant number of healthy hNSCs previously stained with the ∆Ψ-dependent MitoTracker Deep Red (hNSC-Mito) and co-cultured as reported in (**B**). After 24 h, co-cultures were analyzed for the levels of ROS production in **d**SH-SY5Y (**C**) and the percentage of apoptotic **d**SH-SY5Y by assessing caspase 3/7 activity (**D**). The quantitative analysis of ROS production and the measure of apoptotic **d**SH-SY5Y shows that hNSCs strongly reduced ROS production and apoptosis in OGD/R **d**SH-SY5Y only when cells are in direct contact with hNSCs. Data are presented as mean ± SEM or as percentage. Data were obtained using three different hNSC donors. *p = 0.0132; ****p < 0.0001.

We assessed whether ischemia and ischemia/reperfusion compromised mitochondrial activity in human **d**SH-SY5Y. We exposed human **d**SH-SY5Y to 0.2% O_2_ in a glucose- and serum-free medium for 24 h, inducing oxygen-glucose deprivation (OGD). Following this ischemic insult, OGD **d**SH-SY5Y was washed and regenerated in hNSC-medium under normoxic conditions for 24 h (OGD/R). We stained both normoxic, OGD, and OGD/R **d**SH-SY5Y with Mitotracker Green AM, indicating mitochondria mass, and ΔΨ-dependent MitoTracker Red CMXRos, which selectively stained active and functional mitochondria. The Mitotracker Red/Green fluorescent ratio served as a semi-quantitative analysis of mitochondria activity [54, 55]. The data clearly showed that OGD and OGD/R significantly reduced mitochondrial activity, as indicated by the lowest Mito red/green signal observed in OGD and OGD/R dSH-SY5Y compared to those measured in normoxia. Notably, OGD/R cells also displayed cell death morphology (Fig. 6A). This suggests that OGD/R strongly affects mitochondrial activity and can trigger cell death. Since it is known that ischemia/reperfusion induces the excessive production of reactive oxygen species (ROS) and the activation of caspase 3/7-dependent apoptosis [35, 36], we measured ROS production and caspase 3/7 activity.

With the aim to investigate whether hNSCs rescue post-ischemic **d**SH-SY5Y from ROS-induced apoptosis and to evaluate the contribution of paracrine signals and direct-contact dependent mechanisms, OGD **d**SH-SY5Y were stained with DiI, exposed to the ischemic insult, washed, and regenerated in hNSC-medium under normoxic conditions for 24 h (OGD/R), with or without a constant number of healthy hNSCs previously stained with fixable ΔΨ-dependent MitoTracker Deep Red in contact and non-contact co-cultures (Fig. 6B). After 24 h of regeneration, we analyzed **d**SH-SY5Y for ROS production and apoptosis. The data revealed that OGD/R induced significant ROS production and apoptosis in **d**SH-SY5Y. Notably, direct contact with healthy hNSCs substantially reduced ROS production and the percentage of apoptotic **d**SH-SY5Y, effectively restoring the **d**SH-SY5Y to a normoxic state. In non-contact co-culture, the reduction in ROS production and apoptotic **d**SH-SY5Y was only partial (Fig. 6C, D). Similar results were obtained by measuring the cells positive for the cell-impermeant viability indicator ethidium homodimer-1 (EthD-1) (Supplementary Fig. 3). These results indicate that direct contact between hNSCs and post-ischemic **d**SH-SY5Y is essential for the complete rescue of human ischemic **d**SH-SY5Y from ROS-triggered apoptosis.

**Fig. 7:**
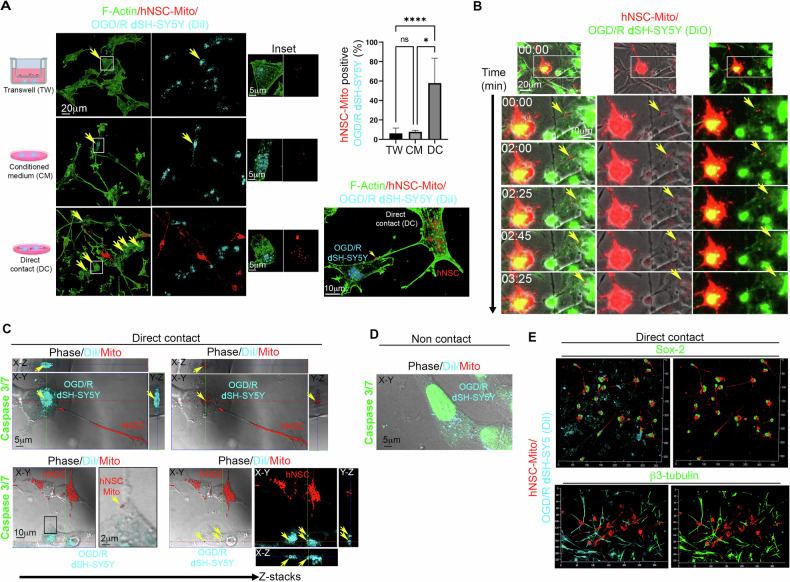
TNT-mediated intercellular transfer of functional mitochondria from healthy hNSCs to OGD/R dSH-SY5Y. **A** The intercellular transfer of functional mitochondria between healthy hNSCs and OGD/R dSH-SY5Y was evaluated using non-contact transwell-based coculture (TW), conditioned medium (CM), or direct contact (DC). Donor hNSCs were stained with ΔΨ-dependent MitoTracker Deep Red (red), acceptor OGD/R dSH-SY5Y were stained with DiO (green), and F-actin staining (cyan) was performed using a cell-permeable CellMask actin, avoiding cell permeabilization. The percentage of receiving OGD/R dSH-SY5Y containing mitochondria from hNSCs was measured in TW, CM, and DC. Only the direct contact coculture allows a robust transfer of mitochondria from donor hNSCs to acceptor OGD/R dSH-SY5Y, while CM allows only rare and isolated mitochondrial signals inside receiving cells. Data are presented as mean ± SEM or as percentage. Data were obtained using three different hNSC donors. In direct contact, we found heterotypic TNTs in which functional mitochondria run from hNSCs to OGD/R **d**SH-SY5Y. *p < 0.05; ****p < 0.0001. Representative images were obtained from three different hNSC donors. **B** Live-cell time-lapse fluorescence microscopy revealed functional mitochondria traveling through TNTs from donor-healthy hNSCs to acceptor OGD **d**SH-SY5Y stained with DiO, as indicated by the yellow arrows. (Supplementary Video 11–13). **C** Direct-contact coculture stained for caspase 3/7 activity and analyzed by confocal microscopy. The *X*–*Y* analysis demonstrated the presence of TNTs containing functional mitochondria connecting hNSCs with OGD-**d**SH-SY5Y and the *Z*-projections clearly show mitochondria originating from donor hNSCs (red, yellow arrows) inside OGD/R **d**SH-SY5Y which was negative for caspase 3/7 activity. **D** Non-contact coculture stained for caspase 3/7 activity and analyzed by *X*–*Y* confocal microscopy at high magnification. **E** The direct contact co-culture was analyzed for stemness and neuronal markers. In this direct-contact coculture hNSCs and OGD/R dSH-SY5Y preserved the expression of stemness (Sox-2) and neuronal (β3-tubulin) markers, respectively. Representative images and data were obtained from three different hNSC donors.

To investigate the functional role of heterotypic TNTs-mediated intercellular mitochondria transfer in post-ischemic neuroprotection, we analyzed the direct-contact coculture, the transwell-based non-contact co-culture between OGD/R **d**SH-SY5Y and healthy hNSCs (TW) and OGD/R **d**SH-SY5Y incubated with hNSCs-derived conditioned-medium (CM). In these experiments, hNSCs were stained with MitoTracker DeepRed and OGD/R **d**SH-SY5Y with DiI, and F-actin staining was performed using a cell-permeable CellMask actin, avoiding cell permeabilization. The number of DiI-receiving cells containing mitochondria was measured in TW, CM, and direct contact (DC). Results show that only the direct contact coculture allows a robust transfer of mitochondria from donor hNSCs to acceptor OGD/R **d**SH-SY5Y (Fig. 7A, arrows). In direct contact, we found heterotypic TNTs in which functional mitochondria ran from hNSCs to OGD/R **d**SH-SY5Y. The live-cell time-lapse fluorescence microscopy analysis of direct contact co-culture showed that hNSCs are strongly connected with OGD/R **d**SH-SY5Y through TNTs, and mitochondria were actively transferred from hNSCs to OGD/R **d**SH-SY5Y (Fig. 7B, Supplementary Videos 11–13). To preserve fragile TNTs and DiI signals, we stained cells with CellEvent™Caspase-3/7Green detection reagent, fixed the direct-contact co-culture, and analyzed it through confocal microscopy without cell permeabilization. The analysis largely confirmed the presence of TNTs containing functional mitochondria that connected hNSCs and OGD/R dSH-SY5Y, the transfer of functional mitochondria from hNSCs to OGD/R dSH-SY5Y, and the absence of caspase 3/7 activation in cells that received functional mitochondria from hNSCs (Fig. 7C, D). In this direct-contact coculture, we found that hNSCs and OGD/R dSH-SY5Y preserved the expression of stemness (Sox-2) and neuronal (β3-tubulin) markers, respectively (Fig. 7E). Collectively, these data demonstrate the central role of heterotypic TNTs and TNTs-mediated mitochondria transfer from healthy hNSCs to post-ischemic **d**SH-SY5Y. This contributes to achieving an efficient rescue of post-ischemic **d**SH-SY5Y from ROS-induced apoptosis.

### hNSCs functionally rescue OGD/R dSH-SY5 through direct-contact-dependent mechanisms

**Fig. 8:**
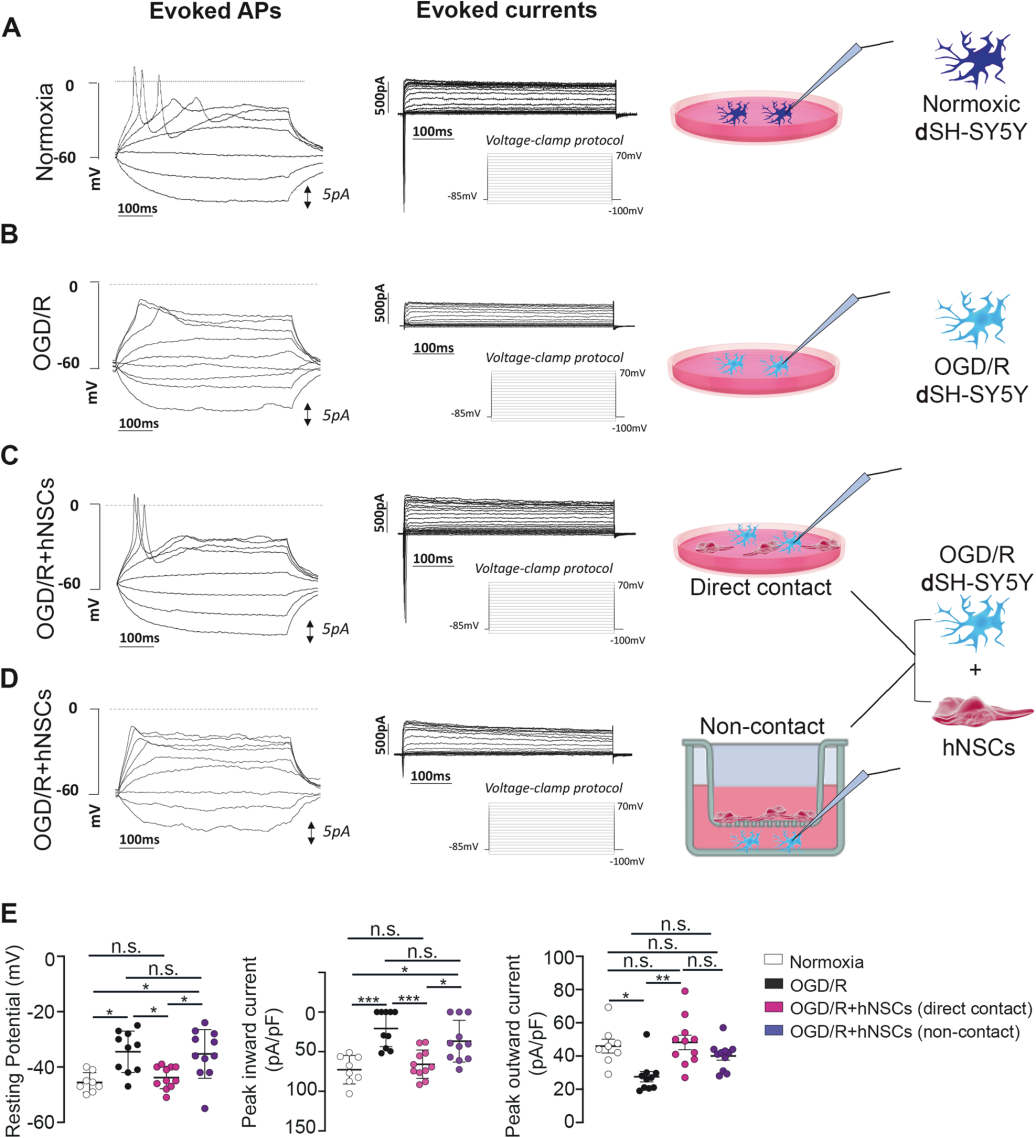
The direct contact with hNSCs is essential for the complete rescue of evoked action potentials, resting potential, and evoked inward current in post-ischemic dSH-SY5Y. **A** Normoxic differentiated SH-SY5Y (**d**SH-SY5Y) was analyzed by patch clamp whole-cell experiments. Normoxic **d**SH-SY5Y generates evoked action potentials (Evoked APs) and shows both are evoked inward current and a sustained evoked outward current (evoked currents). Representative traces are reported. Complete data and statistics are reported in panel **E** and Table 1. **B** OGD/R **d**SH-SY5Y are not able to generate evoked action potentials and show a complete loss of the inward current or a significantly decreased inward current and a significant decrease in the outward current. Representative traces are reported. Complete data and statistics are reported in panel E and Table 1. **C** OGD/R **d**SH-SY5Y cocultured in direct contact with hNSCs were almost completely rescued. In this condition, OGD-R **d**SH-SY5Y exhibited the ability to generate evoked action potentials in 72.7% of analyzed cells and a conspicuous inward current in all cells analyzed, comparable with the control normoxic condition. Representative traces are reported. Complete data and statistics are reported in panel **E** and Table 1. **D** OGD/R **d**SH-SY5Y cocultured non-contact with hNSCs were not able to generate evoked action potentials in the 63.7% of analyzed cells and the inward current evoked remained not fully rescued. Representative traces are reported. Complete data and statistics are reported in panel **E** and Table 1. **E** Graphs and statistics of **d**SH-SY5Y resting potentials, peak inward and outward current in normoxia, OGD/R, OGDR+hNSCs in direct contact, and OGD/R+hNSCs in non-contact coculture are reported. *n* = 8 for normoxic **d**SH-SY5Y and *n* = 11 for other conditions. Data are presented as single data and mean ± SEM. Coculture data were obtained using three different hNSC donors. *p < 0.05; **p < 0.005; ***p < 0.0005.

To investigate whether hNSCs can also functionally rescue post-ischemic **d**SH-SY5Y and evaluate the contributions of paracrine signals and direct-contact-dependent mechanisms, patch clamp whole-cell experiments were conducted. Healthy normoxic **d**SH-SY5Y showed the ability to generate evoked action potentials as a consequence of the expression of both a fast, large inward Na^+^ current and a sustained outward K^+^ current (Fig. 8A–E, Table 1) and showed a membrane potential of about −45 mV (Fig. 8E, Table 1). On the other hand, OGD/R **d**SH-SY5Y were unable to generate evoked action potentials and displayed a significant decrease or a complete absence of the evoked inward current, along with a significant decrease in the evoked outward current (Fig. 8B–E, Table 1). Furthermore, the resting membrane potential was found to be significantly depolarized compared to normoxic **d**SH-SY5Y (Fig. 8E, Table 1).

**Table 1:** Table summarizing data relative to Fig. 8.

	Normoxia	OGD/R	OGD/R + hNSCs Direct contact	OGD/R + hNSCs Non-contact
Resting potential (mV)	−45.57 ± 1.44	−34.50 ± 2.36	−43.82 ± 1.212	−35.27 ± 2.66
Cells with evoked APs (no. of cells/total cells, (%))	8/8 (100%)	2/10 (20%)	8/11 (72.7%)	4/11 (36.3%)
Cells exhibiting evoked inward current (no. of cells/total cells, (%))	8/8 (100%)	4/10 (40%)	11/11 (100%)	8/11 (72.7%)
Peak inward current (pA/pF)	−72.74 ± 17.97	−20.68 ± 22.94	−65.85 ± 5.40	−36.72 ± 26.31
Peak outward current (pA/pF)	45.96 ± 4.78	27.54 ± 3.11	48.18 ± 4.35	39.94 ± 2.27

*n* = 8 for normoxic differentiated SH-SY5Y (**d**SH-SY5Y) and *n* = 11 for other conditions. Data are presented as mean ± SEM or as percentage. Coculture data were obtained using three different hNSC donors. Statistics are reported in Fig. 8E.

When OGD/R **d**SH-SY5Y were co-cultured in direct contact with hNSCs, the resting membrane potential and the inward sodium current were rescued to values similar to the control normoxic condition (Fig. 8E, Table 1). Remarkably, under these experimental conditions, 70% of the analyzed cells were able to generate evoked action potentials (Fig. 8C, Table 1). Conversely, when OGD/R **d**SH-SY5Y were co-cultured with hNSCs through a non-contact coculture system, the rescue of OGD/R **d**SH-SY5Y was only partial. Specifically, only 30% of the cells generated evoked action potentials (Fig. 8D, Table 1), while the inward current and the resting membrane potential did not significantly change when compared to ODG/R **d**SH-SY5Y (Fig. 8E, Table 1). Notably, the resting potential and the peak inward current were found to be statistically different between OGD/R **d**SH-SY5Y in direct contract with hNSCs with respect to the non-contact coculture (Fig. 8E, Table 1).

These data emphasize the crucial role of direct contact between hNSCs and post-ischemic **d**SH-SY5Y for a complete restoration of neuronal electrical activity.

## Discussion

One of the primary objectives of current research on hNSCs is to uncover the mechanisms behind hNSC-related neuroprotection. In this study, we investigated long-range crosstalk between hNSCs and post-ischemic human neuronal cells, exploring the possibility that TNTs could contribute to this mechanism.

We demonstrated that hNSCs communicate with each other via F-actin and nestin-positive TNTs, through which they transfer functional mitochondria. The existence of a TNT-based network between hNSCs and the discovery that nestin serves as a structural component of these TNTs, which transfer functional mitochondria, is significant for two reasons. First, the presence and structural composition of TNTs in hNSCs were previously unknown. Second, these findings could shed light on the functional roles of mitochondria and nestin in preserving hNSCs’ stemness properties. Recent studies have shown that mitochondria influence stem cell maintenance and the cell differentiation process, with mitochondrial pathways found to control stem cell functions [56–58]. This suggests the potential for TNTs-mediated crosstalk in regulating hNSCs’ physiology and stem-related properties. This is in line with recent evidence from mesenchymal stem cells (MSCs) [59, 60].

Using STED super-resolution microscopy, we have uncovered how nestin contributes to TNT structure. We identified a well-ordered assembly state of nestin inside TNTs, forming discrete structures in 3D neurospheres and hNSCs in 2D culture. Using *X*–*Y*–*Z* Tau-STED analysis followed by 3D reconstruction and *Z*-projections, we demonstrated that nestin clusters localize between F-actin fibers, serving as a “bridge”, a localization consistent with a structural function typical of intermediate filaments. Our data are consistent with evidence that nestin is part of the intermediate filament network by copolymerizing with other type III or IV intermediate filament proteins [61, 62].

We speculate that TNTs between hNSCs in 3D neurospheres, as demonstrated here, could contribute to maintaining stem-related properties in hNSCs cultured in vitro. In this context, nestin may play a functional role in TNT assembly, which, in turn, facilitates the intercellular transfer of pro-stemness signals. This hypothesis aligns with evidence indicating that nestin actively contributes to preserving stem-related properties [63, 64].

The second and main aspect addressed in our study is whether our preparation of non-immortalized human neural stem cell lines isolated from brain tissue samples extracted from fetuses that died due to spontaneous abortion is potentially useful for post-ischemic treatments aimed at rescuing neuron functions.

Generally, the ischemic brain contains two different damaged areas: the ischemic core and the penumbra. In the ischemic core, the blood flow is too low, leading to mainly irreversible cell damage and necrosis, while the penumbra offers the potential for salvaging post-ischemic neurons as the ischemic cascade progresses with time, presenting both pro-apoptotic and anti-apoptotic signals [65]. For these reasons, current treatment strategies for ischemic stroke involve rescuing the penumbra through stem cell transplantation, which can shift the balance toward cell recovery [66–69]. Various approaches in the current literature have sought to elucidate the role of long-range crosstalk mechanisms between NSCs and stressed host tissues. This has led to the postulation of the “paracrine hypothesis” or the “bystander effect” as the main hNSCs-dependent neuroprotective mechanisms involved [16, 18, 19].

The experimental model used here aimed to mimic the mechanisms that occur in the ischemic brain when hNSCs are transplanted in the reperfusion phase, acting on the penumbra, with the aim of separating the contribution of paracrine signals and the additive contribution of contact-dependent mechanisms, focusing our attention on heterotypic TNTs.

We provide evidence that TNTs-mediated crosstalk strongly contributes to rescuing human neuronal cells from ischemia-triggered ROS-sustained apoptosis. We observed that ROS and Caspase 3/7 activity were fully restored to normoxic levels only when hNSCs are in direct contact with post-ischemic differentiated SH-SY5Y (**d**SH-SY5Y), while non-contact coculture allows only partial recovery. Finally, plasma membrane resting potentials, evoked action potentials, and evoked currents of post-ischemic **d**SH-SY5Y are almost completely restored to normoxic levels only when hNSCs are in direct contact with ischemic **d**SH-SY5Y, in line with protection from ROS-triggered apoptosis.

Notably, for the non-contact coculture experiments, we used a Transwell® system with a 400 nm pore size, allowing the passage of molecules, exosomes (30–150 nm), and small macrovesicles (100–1000 nm) [70] from hNSCs to ischemic **d**SH-SY5Y and vice versa. We found that when only paracrine signals occurred, the rescue of post-ischemic **d**SH-SY5Y was not complete. This indicates that paracrine signals are important but not sufficient, and heterotypic TNTs and TNTs-dependent mitochondria transfer are necessary for complete neuron rescue. The evidence aligns with the recently proposed “generator hypothesis” [71], wherein cells primarily rely on their own mitochondria for energy metabolism and import and utilize a minimal number of healthy mitochondria from other cells to address metabolic emergencies.

Our findings align with previous reports by Jäderstad and colleagues, who recently found that hNSCs rescue mouse neurons in vivo through a contact-dependent mechanism based on gap-junction formation [72]. It was also shown that gap junctions localize at the tip of closed-ended TNTs between human neurons, and these closed-ended TNTs can evolve into open-ended ones, allowing for the intercellular transfer of cellular cargoes such as mitochondria [73].

Data recently published revealed that direct contact between hNSCs and ischemic neurons reciprocally changes the transcriptome of both cell types, suggesting that deep crosstalk mechanisms occur [74]. Considering that TNTs can transfer RNAs [75] and probably RNA-regulating factors, the TNTs-mediated crosstalk between healthy hNSCs and injured human neuronal cells observed here could play a role in coordinating the transcriptomic modification of both cell types.

Overall, our findings could contribute to elucidating how hNSCs interact with each other and with damaged cells when engrafted into the host’s injured CNS. Furthermore, we provide evidence that non-immortalized hNSC lines isolated from fetal brain tissue are highly effective in rescuing human neuronal cells from loss of function after ischemic stroke.

## Materials and methods

### Human neural stem cell isolation and culture

To produce the hNSCs lines starting from the fetal brain specimen, two main steps have been performed: (i) primary culture and (ii) intermediate product preparation and cryopreservation. (i) Primary culture. Human brain tissue was immediately transferred under strict sterile conditions to the GMP facility in a controlled environment. Then brain specimen was washed in a PBS solution (Dulbecco’s PBS 1X, Carlo Erba Reagent) supplemented with 50 μg/ml of gentamicin and dissociated to reach a monocellular suspension mechanically. Cells were seeded at a density of 10^4^ cells/cm^2^ in a chemically defined culture medium with EGF 20 ng/ml and bFGF 10 ng/ml as previously described [76]. Cultures were maintained in a humidified incubator at 37 °C, 5% O_2_, and 5% CO_2_ and cells were allowed to proliferate as free-floating clusters named neurospheres according to ‘neurosphere assay technique’. This technique allows us to obtain a hNSCs culture with the very same characteristic independently from donor gestation age. Approximately 7–10 days after the primary cell seeding, neurospheres were collected in 15 mL tube, centrifuged, the supernatant discharged, and the cell pellet mechanically dissociated using p200 micropipette. The obtained single-cell suspension was stained with 0.4% Trypan Blue stain solution (Invitrogen, cat #T10282) and counted using a Burker chamber. A cell viability near 95–98% was measured in each neurospheres passage. Throughout these passages, aliquots of cells were frozen as neurospheres and cryopreserved in a culture medium with 10% dimethyl sulfoxide [52]. When adhesion culture was necessary glass supports were coated with Cultrex (BME, Pathclear cat #3432-005-01) 1:50 in hNSC-medium for 1 h at 37 °C in according to manual instruction. In the monoculture experiments, a seeding density of 1 × 10^4^ cells/cm^2^ was used. In both homotype and heterotype co-culture experiments, a ratio of 1:1 was respected, seeding a total of 2.5 × 10^4^ cells/cm^2^.

### SH-SY5Y cells culture and differentiation

Human SH-SY5Y neuroblastoma cells (ATCC, CRL-2266™) were cultured in Dulbecco’s modified Eagle’s medium (DMEM F-12, Invitrogen) supplemented with 10% fetal bovine serum, 100 U/ml penicillin (Invitrogen) and 0.1 mg/ml streptomycin (Invitrogen) at 37 °C in a humidified incubator with 5% CO_2_.

For the experiments, the cover glasses were pretreated with Cultrex™ and cells were seeded at a density of 2.5 × 10^4^ cells per 12 mm (⌀) cover glass in 24-well plates or at the density of 5 × 10^4^ cells per 15 mm (⌀) cover glass in 12-well plates. Immediately after the adhesion of the cells the culture media was substituted with the neuron differentiation medium which consisted of Dulbecco’s modified Eagle’s medium (DMEM F-12, Invitrogen) supplemented with 1% fetal bovine serum, 100 U/ml penicillin (Invitrogen), 0.1 mg/ml streptomycin and 1 μM retinoic acid (RA, S-R2625-, Sigma) [77, 78]. The cells were differentiated for 7 days before their use, with the differentiating medium being replaced daily. The RA was freshly added to the medium immediately before the use. The correct differentiation of human SH-SY5Y was evaluated by whole-cell patch clamp experiments as the ability to generate evoked action potentials [77, 78]. Correctly differentiated human SH-SY5Y was indicated in the main text as **d**SH-SY5Y.

### Immunofluorescence in neurospheres and 2D-culture

Neurospheres at 7–10 days were collected, resuspended in PBS, and fixed in 4% paraformaldehyde for 30 min. After fixation, neurospheres were washed in PBS and cryoprotected in 30% sucrose in PBS overnight at 4 °C. The day after, neurospheres were gently collected by centrifugation, and the sucrose/PBS solution was removed. Fixed and cryoprotected neurospheres were resuspended in OCT-medium, frozen in liquid nitrogen, and cryosection in 50 μm depth sections were prepared. Sectioned neurospheres were permeabilized by 0.3% Triton X-100 in PBS for 30 min, blocked in 3% BSA, 0.3% Triton X-100 in PBS for 30 min, and incubated with primary antibody diluted in 0.3% Triton X-100, 3% BSA in PBS for 24 h at 4 °C. The day after, they were extensively washed with 1.5% BSA, 0.15% Triton X-100 in PBS and incubated in the same solution with secondary antibodies for 24 h. The day after, sections were washed many times in PBS and mounted in Vectashield antifade mounting medium (Vector Laboratories cat. #H-1000-10) for confocal microscopy.

In 2D culture, to minimize breakage of the TNTs, fixation with 4% paraformaldehyde was performed for 10 min at room temperature, gently replacing the culture medium with it. Afterward, samples were carefully washed with PBS, permeabilized by 0.3% Triton X-100 in PBS for 15 min, blocked with 3% BSA in PBS for 30 min, and incubated with primary antibody in 3% BSA in PBS for 24 h at 4 °C. After the incubation the samples were gently washed with 3% BSA in PBS multiple times and then incubated with secondary antibodies for one hours at room temperature in 3% BSA in PBS, finally washed many times and mounted in Vectashield antifade mounting medium (Vector Laboratories cat. #H-1000-10).

### Antibodies and working dilution

CD15/SSEA1 (MC480) Mouse mAb (Cell Signaling, cat #4744) 1:500. SOX2 (L1D6A2) Mouse mAb (Cell Signaling, cat #4900) 1:400. Nestin (10C2) Mouse mAb #33475 (Cell Signaling, cat #33475) 1:1000. Musashi-1 Antibody (R&D Systems, cat #AF2628) 1:100.

Donkey anti-mouse IgG (H + L) highly cross-adsorbed secondary antibody, Alexa Fluor™ 488 (Thermo Fisher, cat. #A-21202) 1:1000. Donkey anti-Goat IgG (H + L) highly cross-adsorbed secondary antibody, Alexa Fluor™ 546 (Thermo Fisher, cat. #A-11056) 1:1000.

### Electron microscopy

For transmission electron microscopy (TEM), after 12 days in culture hNSCs were collected, washed in PBS 1X (w/o Ca/Mg), and fixed with 2.5% glutaraldehyde in 0.1 M PBS, pH 7.4, at 4 °C for 2 h, washed, post‐fixed with 1% OsO_4_ at 4 °C for 1 h. After rinsing in 0.1 M PBS, the samples were dehydrated in graded concentrations of acetone and embedded in a mixture of Epon and Araldite (Electron Microscopic Sciences, Fort Washington, PA, USA). Ultrathin sections were cut at 70 nm thickness on an Ultracut E ultramicrotome (Reichert-Jung, Heidelberg, Germany), contrasted with lead citrate, and observed on a Philips Morgagni 268 D electron microscope (Fei Company, Eindhoven, The Netherlands), equipped with a Megaview III camera for acquisition of digital images.

For scanning electron microscopy (SEM) analysis, after 12 days in culture hNSCs were collected, washed in PBS 1X (w/o Ca/Mg), and fixed in 2,5% glutaraldehyde in 0.1 M PB for 2 h at 4 °C, washed in the same buffer, post-fixed with 1% OsO_4_ at 4 °C for 1 h, and dehydrated with graded concentrations of ethanol (Fluka). The samples were then treated using a drying agent hexamethyldisilazane (HMDS), mounted on metallic specimen stubs, and sputter-coated with gold (MED 010 Balzers). SEM imaging was performed by an XL30 ESEM (FEI-Philips).

### Staining of mitochondria, F-actin and plasma membrane for live-cell fluorescence microscopy

For live-imaging, neurospheres were incubated in suspension with 1uM CellMask™ Green Actin Tracking Stain (Invitrogen, cat. #A57243) diluted in hNSCs medium for 30 min at 37 °C. After 30 min of incubation, cells were centrifugated and resuspended in fresh prewarmed hNSC-medium at least two times. The day after we were seeded on the confocal dish (Greiner Bio-One, cat. #627870) previously coated with the cultrex as described above.

For live imaging of mitochondria in the neurosphere, spheres were incubated with 100 nM Mito-Tracker Green FM (MT; Invitrogen, cat. #M7514) or 100 nM MitoTracker™ Red CMXRos (Invitrogen, cat. #M7512) diluted in hNSC-medium. After 30 min of incubation, cells were centrifugated and resuspended in fresh prewarmed hNSC-medium at least two times, left overnight in culture, and centrifugated. The cell pellet was washed once to clear the excess dye completely. The day after were seeded on a confocal dish previously coated with cultrex as described above.

For live-imaging of mitochondria, hNSCs single-cell suspension was incubated with 100 nM Mito-Tracker Green FM (MT; Invitrogen, cat. #M7514) or with 100 nM MitoTracker™ Red CMXRos (Invitrogen, cat. #M7512) diluted in hNSC-medium. After 30 min of incubation, cells were centrifugated and resuspended in fresh prewarmed hNSC-medium at least two times, left overnight in culture, and centrifugated. The cell pellet was washed once to clear the excess dye completely and seeded on cultrex-coated glass. In coculture experiments, the plasma membrane of receiving cells was stained with the Vybrant™ Multicolor Cell-Labeling Kit using DiO or DiI dyes (Thermofisher Catalog number: V22889) at a 1:500 dilution for 30 min.

### Staining of mitochondria, F-actin, and plasma membrane for fixed-cell-based microscopy analysis

For fixed-cell-based analysis, single-cell suspension of hNSCs was incubated with 100 nM Mito-Tracker Deep-Red FM (MT; Invitrogen, cat. #M22426) diluted in hNSC-medium. After 30 min of incubation, cells were centrifuged e resuspended in fresh prewarmed hNSC-medium at least two times, left overnight in the hNSC-medium and centrifuged. The cell pellet was washed once to clear the excess dye completely and seeded on cultrex-coated glass. For the preparation of hNSCs conditioned medium, cells were stained and seeded as previously reported, and the medium was collected after 24 h of incubation. This medium was used as a conditioned medium.

To reduce the breakdown of TNT and maintain the fluorescence of MitoTracker Deep-Red, cells were fixed directly in the medium by adding formaldehyde to a final concentration of 3.7% v/v for 15 min at 37 °C. Afterward, samples were carefully washed with PBS, permeabilized by 0.3% Triton X-100 in PBS for 15 min, blocked with 3% BSA in PBS, and incubated with AlexaFluor488- Phalloidin (1:500; Thermo Fisher, cat. #A12379) for 60 min at room temperature in order to stain F-actin. After careful washing, the samples were mounted in Vectashield antifade mounting medium (Vector Laboratories cat. #H-1000-10). In some experiments, to avoid cell permeabilization, F-actin was stained using CellMask Actin Tracking Stain (ThermoFisher, cat. #A57249) according to the manufacturer’s instructions. In coculture experiments, the plasma membrane of receiving cells was stained with the Vybrant™ Multicolor Cell-Labeling Kit using DiO or DiI dyes (Thermofisher Catalog number: V22889) at a 1:500 dilution for 30 min.

### Live-cells time-lapse fluorescence microscopy

For live-cell imaging, single cells or neurospheres were seeded on confocal dishes (Greiner Bio-One, cat. #627870), and images were acquired by Biostation IM-Q at 37 °C, humidity level of 95 and 5% CO_2_. To correctly visualize the mitochondria’ dynamics, images were acquired every 5–7 s with a low-intensity light source to reduce phototoxicity and photobleaching of the dye. Timelapse images were analyzed using NIS ELEMENTS AR software and Fiji software.

### Confocal microscopy and Tau-STED microscopy of hNSCs in 2D-culture and sectioned neurospheres

Routine confocal microscopy was performed using a Leica TCS SP5 microscope. All acquisitions were performed by serial acquisition mode between frames. *XYZ*-series were acquired with a raster size of 1024 × 1024 pixels in the *X*–*Y* planes and a *Z*-step of 0.2 μm between optical slices. For super-resolution Tau-STED microscopy acquisitions Leica Stellaris 8 STED microscope was used.

Three-dimensional (3D) images and projections from z-stack were constructed and processed using Leica Application Suite X software (LASX). Images were analyzed using LASX and FIJI software. The identification of TNTs between human neural stem cells and between hNSCs and differentiated SH-SY5Y was performed as already reported [26].

### Measure of nestin cluster area and TNT-diameter

Super-resolved confocal *X*–*Y* images obtained using Leica Stellaris 8 STED microscope were analyzed for nestin cluster areas using Fiji software. TNTs-diameter were measured using confocal *X*–*Y* images obtained by Leica TCS SP5 microscope through the analysis of F-actin and Nestin fluorescence intensity profile (PSF) followed by the measure of the full width at half-maximum (FWHM).

### Oxygen glucose deprivation/reoxygenation of differentiated SH-SY5Y and coculture with hNSCs

The day before the treatment, glucose- and serum-free DMEM (DMEM no glucose, Gibco cat. #11966025) was conditioned in a hypoxic gas mixture (see below) for 24 h (OGD medium) and differentiated SH-SY5Y were seeded on glass, coated with cultrex 1:50 for 1 h at 37 °C (BME, Pathclear cat #3432-005-01) with a seeding density of 2.5 × 10^4^ cells/cm^2^. The day after seeding, differentiated SH-SY5Y were incubated with 1:500 Vybrant DiI Cell-Labeling Solution (Thermo Fisher, cat. #V22885) in complete growth media for 45 min. Then, cells were washed with glucose- and serum-free DMEM (DMEM no glucose, Gibco cat. #11966025) and left for one hour in an incubator at 37 °C. After this treatment, cells were gently washed with OGD medium, placed in a hypoxic chamber conditioned with 0.2% O_2_, 5% CO_2_, 95% N_2_ (20 L/min for 4 min) in OGD medium, and incubated at 37 °C for 24 h (OGD). Then, the cells were washed and reoxygenated with or without healthy hNSCs previously stained with the ∆Ψ-dependent MitoTracker Deep Red (hNSC-Mito) cocultured in direct contact or non-contact co-culture system. For the non-contact co-culture Corning Transwell® insets with 0.4 μm pore size membrane were used. Cocultures were placed in a normoxic incubator at 37 °C, 5% CO_2_ in hNSCs-medium for 24 h (OGD/R).

### Analysis of mitochondria activity

Normoxic and OGD/R differentiated SH-SY5Y were stained with AM 100 nM Mito-Tracker Green (Invitrogen, cat. #M7514) and 100 nM ΔΨ-dependent MitoTracker Red CMXRos (Invitrogen, cat. #M7512) diluted in hNSC-medium for 30 min, carefully washed and images were acquired using Biostation IM-Q. The Mitotracker Red/Green fluorescent ratio was measured using FiJi software as a semi-quantitative analysis of mitochondria activity [54, 55].

### Quantification of intercellular mitochondrial transfer

The number of DiO- or DiI-receiving cells containing mitochondria from donor hNSCs were measured using LASX software in both homotypic coculture (hNSCs MitoTracker Deep Red/hNSCs DiO) and heterotypic coculture (hNSCs MitoTracker Deep Red/dSH-SY5Y DiI or DiO). Transwell-based non-contact coculture (TW), donor-cell conditioned medium (CM), and direct contact (DC) coculture were analyzed.

### Caspase-3/7 activity assay and viability assay

Cells were incubated with 1 μL of the CellEvent™Caspase-3/7Green detection reagent (Thermofisher, cat. #C10423) added directly in the 24-well in hNSC-medium for 30 min in an incubator at 37 °C, 5% CO_2_. After 30 min, cells were fixed, adding 37% formaldehyde to a final concentration of 3.7% at 37 °C for 15 min. After the fixation cells were gently washed with PBS and immediately analyzed. The quantification of apoptotic differentiated SH-SY5Y was performed by confocal microscopy using Leica TCS SP5 microscope as double positive DiI/Caspase 3-7 positive cells. Random areas were acquired per biological replicate, and apoptotic differentiated SH-SY5Y were counted as a percentage of total differentiated SH-SY5Y. To detect necrotic cells, the cell-impermeant viability indicator ethidium homodimer-1 (EthD-1) was added to the medium at the final concentration of 0.5 μM, and images were acquired using an inverted Nikon Ti-2 microscope.

### Analysis of reactive oxygen species (ROS)

Cells were incubated with 5 μM CellROX Green Reagent (C10444, www.thermofisher.com) in hNSC-medium for 30 min at incubator at 37 °C, 5% CO_2_. After 30 min cells were fixed, adding 37% formaldehyde to a final concentration of 3.7% at 37 °C for 15 min. After the fixation, cells were gently washed with PBS and immediately analyzed by confocal microscopy using a Leica TCS SP5 microscope. ROS production was measured in DiI-positive differentiated SH-SY5Y using FiJi software as mean fluorescence intensity per cell.

### Whole-cell patch clamp analysis of differentiated SH-SY5Y

Whole-cell patch clamp experiments were conducted under the optical guidance of an Olympus B51WI microscope using the Multiclamp 700B amplifier (Axon CNS-Molecular Devices, Sunnyvale, CA, USA) interfaced with the Axon Digidata 1500 (Axon Instrument-Molecular Devices, Sunnyvale, CA, USA). Currents were sampled at 10 kHz and low-pass filtered at 5 kHz. Borosilicate patch pipettes were pulled to achieve tip resistances of 5–7 MΩ with a P-1000 pipette puller (SUTTER INSTRUMENT, Novato, CA 94949, USA) and filled with a pipette solution containing (in mM): 130 K-gluconate, 10 NaCl, 1 CaCl_2_, 1 EGTA, 10 Hepes, 2 ATP-Na_2_, 2 MgCl_2_, with a pH of 7.2 adjusted with KOH and an osmolarity of 280 mmol/kg. The recordings were conducted at room temperature on SHSY5Y cells plated on Cultrex®-coated coverslips (15 mm ⌀) using a bath solution containing (in mM): 140 NaCl, 2.8 KCl, 1 CaCl_2_, 0.01 EDTA, 10 Hepes, with a pH of 7.2 adjusted with NaOH and an osmolarity of 283–284 mmol/kg. The biophysical profiles of four different experimental conditions were investigated: (i) Normoxic differentiated SHSY5Y cells; (II) OGD/R differentiated SHSY5Y cells; (III) OGD/R SHSY5Y differentiated cells cocultured with hNSCs in direct contact; (IV) OGD/R SHSY5Y differentiated cells cocultured with hNSCs in non-contact coculture.

The identification of differentiated SH-SY5Y in the direct contact co-culture was achieved through both a morphological evaluation of the two cell types and DiI fluorescence labeling of the differentiated SH-SY5Y before co-culturing. The co-culture experiments were performed with hNSCs obtained by three different spontaneously aborted fetuses.

Current-clamp experiments, in gap-free mode without current injection, were performed to monitor the resting membrane potential. Additionally, depolarizing steps of current (of 500 ms in duration, with increments of 5 pA) were injected to investigate the cells’ ability to generate action potentials. The criterion used to distinguish an evoked action potential was the presence of the overshoot in the evoked event. A voltage-clamp protocol composed of depolarizing steps of current of 10 mV from −100 to 70 mV, with a holding potential of −85 mV, was applied to investigate the evoked current. The recorded currents were normalized for the cell capacitance.

### Experimental design and statistical analysis

All data represent at least three replicates from three independent hNSC donors. Statistical analyses were conducted using GraphPad Prism 6 software (GraphPad Prism). All data are reported as the mean ± SEM. Statistically significant differences were computed using the Student’s *t*-test for unpaired data and two-way ANOVA with Tukey’s multiple comparisons test for multiple statistical comparisons between groups. The significance level was set at *p* < *0.05*.

### Supplementary information

Supplementary Figures Legends

Supplementary Figure 1

Supplementary Figure 2

Supplementary Figure 3

Video 1

Video 2

Video 3

Video 4

Video 5

Video 6

Video 7

Video 8

Video 9

Video 10

Video 11

Video 12

Video 13

## Data Availability

All data supporting the findings of this study are available within the paper and its Supplementary Information. The materials described in the manuscript, including all relevant raw data, will be freely available to any researcher wishing to use them for non-commercial purposes, without breaching participant confidentiality.
