# Supplementary material for: Human neural stem cells derived from fetal human brain communicate with each other and rescue ischemic neuronal cells through tunneling nanotubes
Source: Cell Death Dis. 2024 Sep 1;15(8):639. doi: 10.1038/s41419-024-07005-w (PMC11365985; doi:10.1038/s41419-024-07005-w)
Supplement: Supplementary file 1 — Supplementary Figures Legends [file 41419_2024_7005_MOESM1_ESM.docx]

**Supplementary Figure 1**

**Evidence of F-Actin and Nestin-Positive TNTs Between hNSCs**

**A-B.** Laser-scanning confocal microscopy (LSCM) and 3D reconstruction analysis of F-actin (in green) and Sox-2 (in blue) localization (A) and F-actin (in green) and Nestin (in blue) localization (B) in hNSCs cultured in a 2D environment. The enlarged insets depict a 3D reconstruction of an F-actin-positive TNT viewed from both an upper and lateral angle in A and nestin-positive TNTs in B. Representative images obtained from three different hNSCs donors.

**C.** 3D-LSCM was employed to specifically analyze TNTs that extended beyond the substrate. The diagram on the left and the corresponding 3D reconstructions on the right demonstrate that TNTs were identified as detached from the glass support. TNTs are clearly visible in the 3D reconstruction using upper optical planes (indicated by the yellow arrow). Representative images obtained from three different hNSCs donors.

**D.** An X-Y slicing analysis and lateral Z-projections of a TNT that is positive for both Nestin and F-actin and extends beyond the substrate, as indicated in panel C. The zoomed inset highlights the co-localization of F-actin and Nestin. The F-actin and Nestin Z-stack full-width at half maximum (FWHM) measurements were used to determine the diameter of both F-actin and Nestin fibers within these TNTs. No differences were observed; both fibers exhibited an average diameter of 550 nm. Data obtained using three different hNSCs donors.

**Supplementary Figure 2**

**hNSCs generate TNTs with normal differentiated SH-SY5Y (dSH-SY5Y)**  **through which they exchange functional mitochondria**

**A.** hNSCs were stained with fixable ΔΨ-dependent MitoTracker Deep Red (red) and cocultured in direct contact or in non-contact with human  **d**SH-SY5Y stained with DiI (cyano). After 24h F-actin was stained and coculture was analyzed by confocal microscopy. Mitochondria were transferred from hNSCs to  **d**SH-SY5Y only in the direct contact coculture (yellow arrows).

**B.** A more detailed analysis of direct contact coculture show heterotypic TNT connecting hNSCs and  **d**SH-SY5Y. Note the presence of mitochondria from hNSCs inside TNT (arrows). The inset shows a deeper analysis of receiving  **d**SH-SY5Y through XY and Z-projections. Note the presence of red mitochondria, that come from

hNSCs, inside TNT and inside neuron (arrows).

Representative images and data obtained from three different hNSCs donors.

**Supplementary Figure 3**

**d**SH-SY5Y cells were cultured under normoxic conditions or exposed to OGD, then washed and reoxygenated (OGD/R) with or without a constant number of healthy hNSCs in direct contact or in non-contact coculture. After 24 hours, cells were analyzed for EthD-1 staining. Representative images and data were obtained from three different hNSCs donors.
