# Supplementary figures and images for: Human neural stem cells derived from fetal human brain communicate with each other and rescue ischemic neuronal cells through tunneling nanotubes
Source: Cell Death Dis. 2024 Sep 1;15(8):639. doi: 10.1038/s41419-024-07005-w (PMC11365985; doi:10.1038/s41419-024-07005-w)

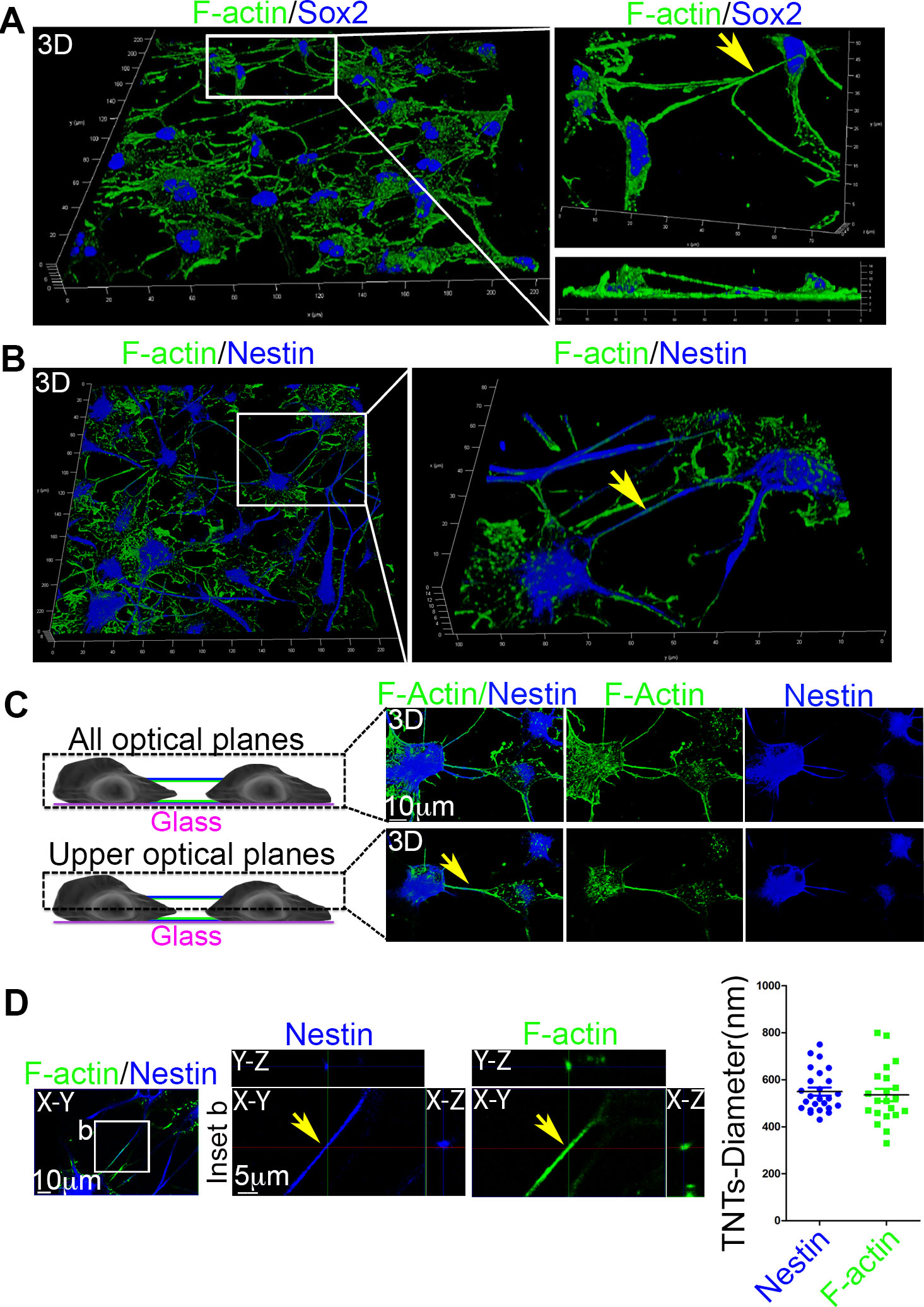

Supplement: Supplementary file 2 — Supplementary Figure 1 [file 41419_2024_7005_MOESM2_ESM.jpg]

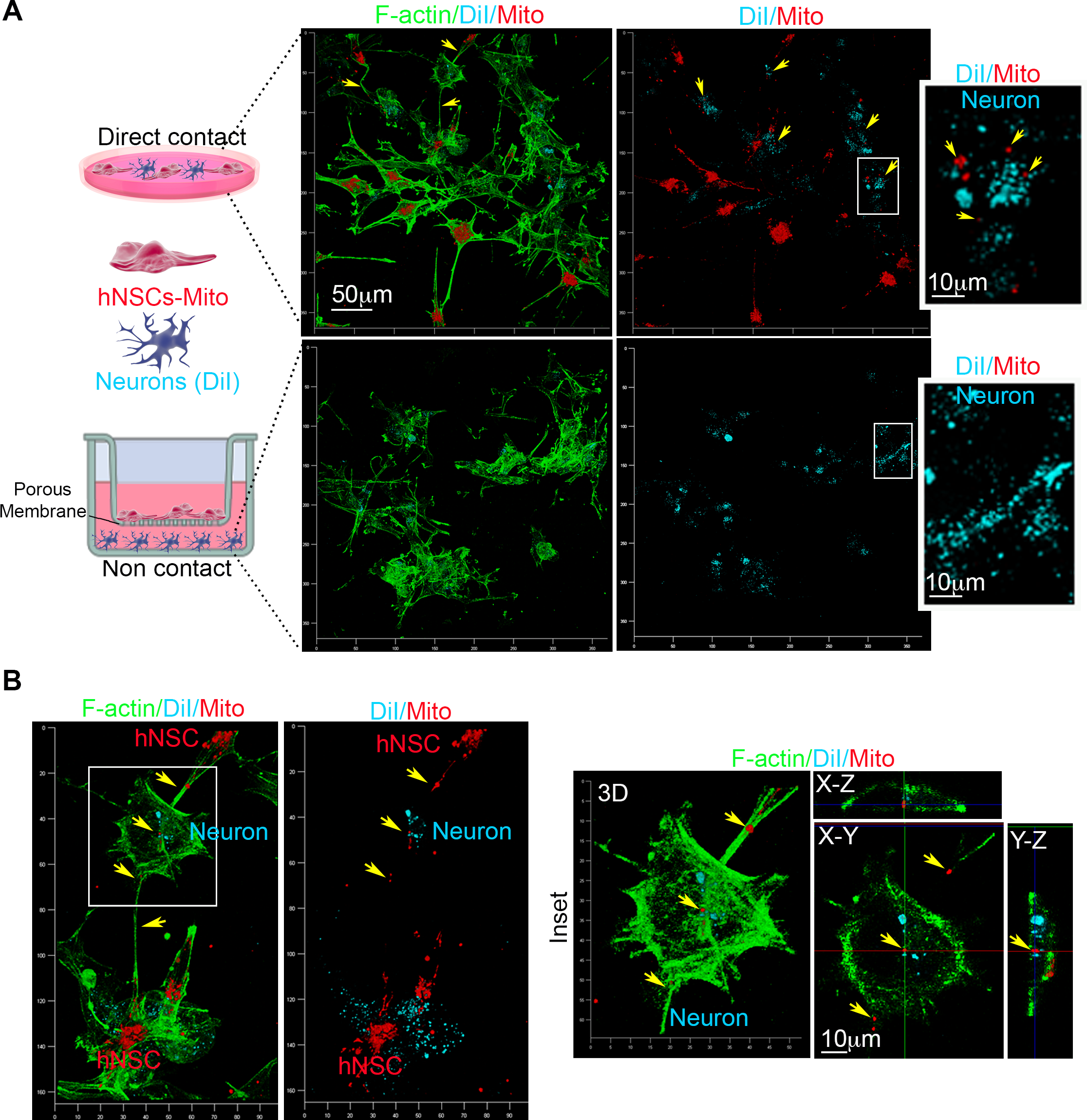

Supplement: Supplementary file 3 — Supplementary Figure 2 [file 41419_2024_7005_MOESM3_ESM.tif]

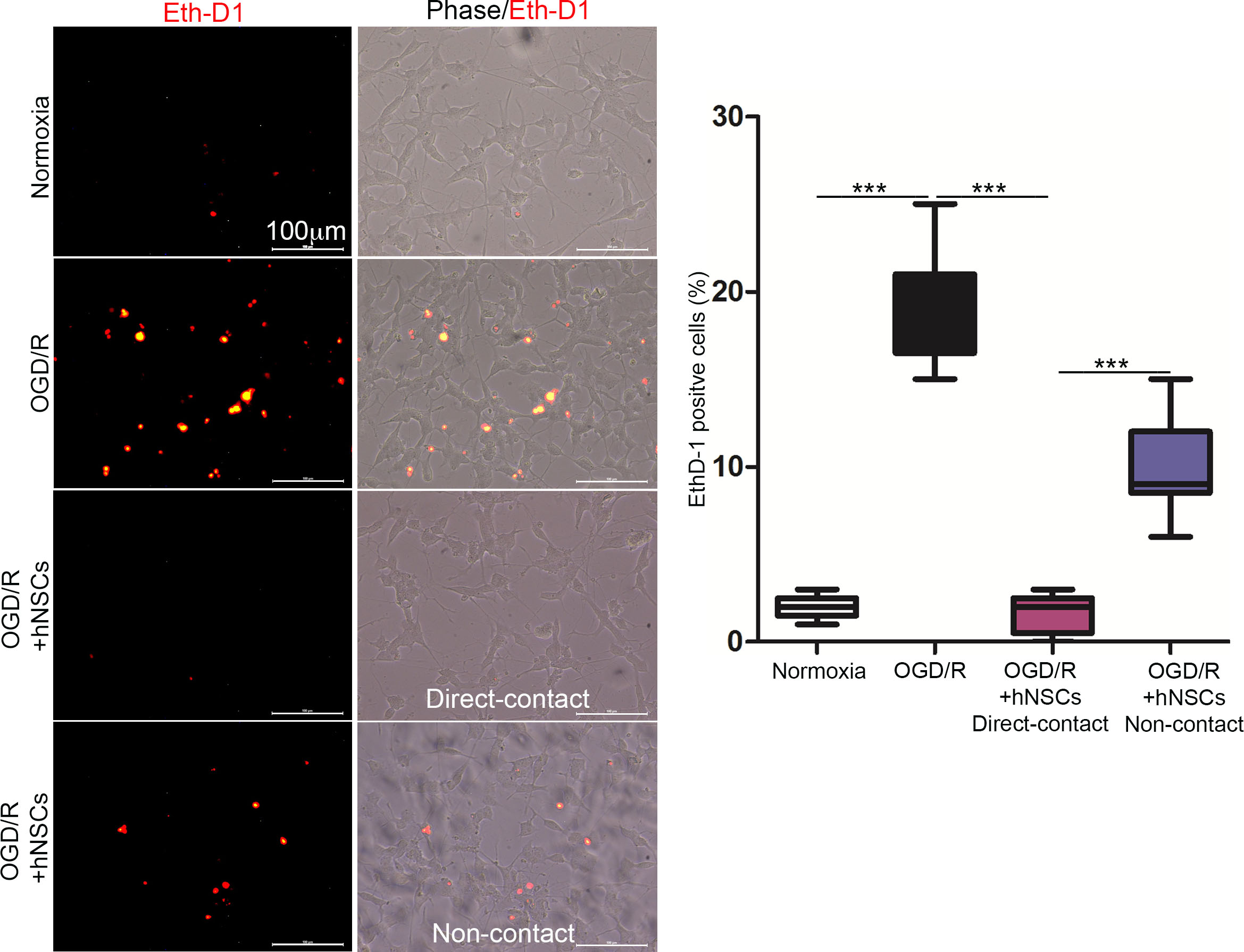

Supplement: Supplementary file 4 — Supplementary Figure 3 [file 41419_2024_7005_MOESM4_ESM.jpg]
